# Neuroprotection Mediated by P2Y_13_ Nucleotide Receptors in Neurons

**DOI:** 10.1016/j.csbj.2015.02.002

**Published:** 2015-02-17

**Authors:** Raquel Pérez-Sen, Mª José Queipo, Verónica Morente, Felipe Ortega, Esmerilda G. Delicado, Mª Teresa Miras-Portugal

**Affiliations:** Biochemistry Department, School of Veterinary Sciences, Complutense University of Madrid, Institute of Neurochemistry (IUIN), Madrid, Spain; Health Research Institute of the Hospital Clinico San Carlos (IdISSC), Spain

**Keywords:** P2Y_13_ receptor, Nucleotide receptors, ERK1/2 signaling, GSK3 signaling, MAPK protein phosphatases, DUSP, Neuroprotection

## Abstract

ADP-specific P2Y_13_ receptor constitutes one of the most recently identified nucleotide receptor and the understanding of their physiological role is currently under investigation. Cerebellar astrocytes and granule neurons provide excellent models to study P2Y_13_ expression and function since the first identification of ADP-evoked calcium responses not attributable to the related P2Y_1_ receptor was performed in these cell populations. In this regard, all responses induced by ADP analogues in astrocytes resulted to be Gi-coupled activities mediated by P2Y_13_ instead of P2Y_1_ receptors. Similarly, both glycogen synthase kinase-3 (GSK3) and ERK1/2 signaling triggered by 2MeSADP in cerebellar granule neurons were also dependent on Gi-coupled receptors, and mediated by PI3K activity. In granule neurons, P2Y_13_ receptor was specifically coupled to the main neuronal survival PI3K/Akt-cascade targeting GSK3 phosphorylation. GSK3 inhibition led to nuclear translocation of transcriptional targets, including β-catenin and Nrf2. The activation of the Nrf2/heme oxygenase-1 (HO-1) axis was responsible for the prosurvival effect against oxidative stress. In addition, P2Y_13_-mediated ERK1/2 signaling in granule neurons also triggered activation of transcription factors, such as CREB, which underlined the antiapoptotic action against glutamate-induced excitotoxicity. Finally, a novel signaling mechanism has been recently described for a P2Y_13_ receptor in granule neurons that involved the expression of a dual protein phosphatase, DUSP2. This activity contributed to regulate MAPK activation after genotoxic stress. In conclusion, P2Y_13_ receptors harbored in cerebellar astrocytes and granule neurons exhibit specific signaling properties that link them to specialized functions at the level of neuroprotection and trophic activity in both cerebellar cell populations.

## 1 Introduction

A growing body of literature strongly supports the involvement of extracellular nucleotides as key messenger molecules modulating important functions at different cellular populations of the nervous system. Nucleotides activate both ionotropic (P2X) and metabotropic (P2Y) receptors [4,6]. Among metabotropic P2Y nucleotide receptors, some of them, such as P2Y_2_ receptors, are well characterized within the nervous system in terms of their coupled intracellular signaling cascades that promote relevant functions related to neuroprotection and neuroregeneration [57]. In fact, correct function and expression of P2Y_2_ receptor seem to be essential for a better outcome in mouse models of neurodegenerative diseases [1,34]. In addition, P2Y_1_ receptors also play important roles in the nervous system, being present at very early stages of the development in radial glia, contributing to their proliferation, migration and subsequent guidance of the cortical neuron population [58].

P2Y_13_ receptor is one of the most recently cloned P2Y receptors. It belongs, together with P2Y_1_ and P2Y_12_, to a distinct structural branch of P2Y receptors specific for adenine diphosphate nucleotides. Likewise, P2Y_12_, P2Y_13_ and the UDP-glucose responding P2Y_14_ receptors, enclose a subfamily of Gi-coupled receptors, which differ from Gq-coupled P2Y_1_ receptors. P2Y_12_ receptors were first cloned and characterized as the target of ADP-stimulated thrombus formation in platelets [24], but they seemed not to be expressed in the brain. Therefore, the receptor responsible for abundance of Gi-linked activity mediated by ADP and 2MeSADP in brain remained unidentified until the cloning of human and murine P2Y_13_ receptors, when it was recognized as the previously known GPR-86 [7,60]. P2Y_13_ receptor shared sequence identity with P2Y_12_ receptor, but they can be distinguished upon different pharmacological and signaling properties [7,39,40]. The identification of the rat P2Y_13_ receptor confirmed the existence of this new member of the Gi-coupled P2Y subfamily [15], which exhibited several differences with respect to human and murine counterparts. The expression profile revealed that it was very abundant in the spleen as well as in the rat brain, suggesting an important role for this receptor in both the immune and nervous systems.

This background data prompted us to study the expression of this kind of receptors in cellular populations of the cerebellar cortex, where a high Gi-linked ADP activity was reported [33]. Accordingly, during the progress of our studies with primary cultures of rat cerebellar astrocytes and granule neurons, we accumulated a great deal of evidence involving specific ADP-mediated signaling non-attributable to other previously characterized nucleotide receptors. These results are covered in detail in separate sections throughout this review, in which we describe the identification of P2Y_13_ receptors in both, purified astrocyte and granule cell cultures, as well as their coupled signaling to main intracellular cascades related to cell maintenance and survival.

## 2 Presence of Functional P2Y_13_ Receptors in Rat Cerebellar Astrocytes

**Fig. 1:**
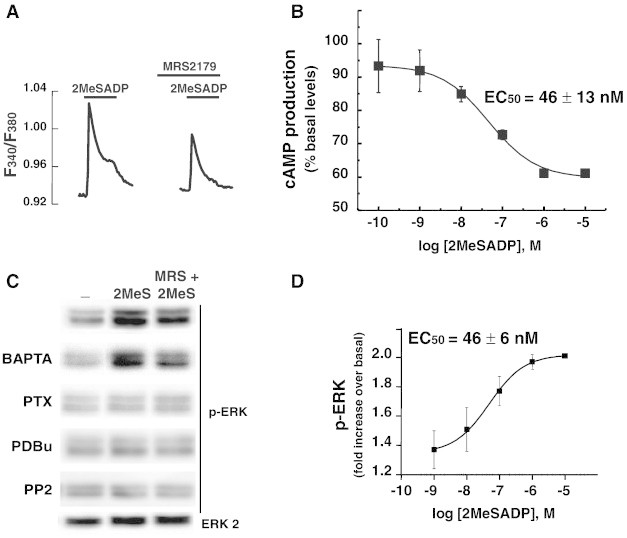
2MeSADP responses in rat cerebellar astrocytes. A. 2MeSADP calcium responses in single cerebellar astrocytes. Cells loaded with fura-2 were challenged with 10 μM 2MeSADP for 30 s and the increases in the fluorescence ratio (F340/F380) calculated as described [5]. Where indicated cells were pre-incubated with 10 μM MRS2179 for 3 min previous to be stimulated with 2MeSADP in the continuous presence of the P2Y_1_ receptor antagonist. B. Concentration response curve obtained for the inhibition of cAMP production induced by isoproterenol. Cells were stimulated for 3 min with 10 μM isoproterenol in the presence or absence of different 2MeSADP concentrations and the cAMP levels were determined by the enzyme-immunoassay from Amersham. C. ERK activation induced by 2MeSADP stimulation in cerebellar astrocytes. The presence of the active form of ERK (phosphor-ERK) was detected by western blot experiments from total cell lysates obtained from astrocytes, which were stimulated for 5 min with 10 μM 2MeSADP. Where indicated astrocytes were preincubated with *Pertussis Toxin* (100 ng/mL, overnight), BAPTA-AM (10 μM, 30 min), PDBU (200 nM, overnight) or PP2 (10 μM, 30 min), previous to be stimulated with the nucleotide. Similar experiments were carried out in cells pretreated with the P2Y_1_ receptor antagonist. The non-phosphorylated form of ERK was also detected. Panel shows the results of a representative experiment of each experimental condition. D. Diagram shows the concentration response curve obtained for the ERK-activation induced by 2MeSADP in rat cerebellar astrocytes. Cells were stimulated for 5 min with different 2MeSADP concentrations and the phosphor-ERK quantified by immunoblotting.

Our studies on P2Y_13_ receptors began in rat purified cerebellar astrocyte cultures, which constituted an excellent model to characterize metabotropic P2Y receptor signaling. In previous works, we described that cerebellar astrocytes expressed a great variety of metabotropic P2Y receptors [27]. The majority of the cells co-expressed at least two functional P2Y subtypes, P2Y_1_ and P2Y_2/4_ receptors, at that time these receptors were activated with 2MeSATP and UTP, respectively [27]. Besides, 2MeSATP and UTP evoked calcium responses were strongly potentiated by the co-stimulation of Gs-coupled receptors co-expressed in the same cell, such as ligands of beta-adrenoceptors, as well as by other purinergic compounds including adenosine or the dinucleotide Ap_5_A [28]. These results indicated that purinergic signaling played an important role in these glial cells. Later on, the availability of new agonist for ADP receptors, the non-hydrolyzable analogue, 2MeSADP, and the identification of new subtypes P2Y_12/13_ receptors, allowed us to re-characterize P2Y_1_ responses in cerebellar astrocytes. We confirmed that all individual astrocytes responded to 2MeSADP stimulation with calcium responses similar to that observed with 2MeSATP, but surprisingly they exhibited a different sensitivity to the specific P2Y_1_ antagonist, MRS2179. Unexpectedly, most astrocytes exhibited 2MeSADP induced calcium responses in the presence of the P2Y_1_ antagonist, and only a small population of astrocytes, accounting only for 13% of tested cells, did not display calcium responses in the presence of the antagonist, which corresponded to cells that only expressed functional P2Y_1_ receptors. A 38% of tested cells exhibited 2MeSADP evoked calcium responses insensitive to MRS2179 antagonism and a 49% of the cells were partially sensitive, indicating that another ADP receptor was present in these cells (Fig. 1A). Taking into account that the new ADP receptors were Gi-coupled receptors, their presence and functionality were investigated by analyzing the effect of ADP and 2MeSADP on cAMP production induced by isoproterenol. These experiments were performed in the presence of MRS2179 and adenosine deaminase to avoid any possible interference with P2Y_1_ receptor or A_2B_ adenosine receptors, also present in these glial cells (Fig. 1B). The pharmacological profiles of responses inhibiting isoproterenol-induced cAMP production, the sensitivity to *Pertussis Toxin* and the insensitivity to P2Y_12_ receptor antagonists revealed that the functional Gi-linked ADP receptor present in cerebellar astrocytes was a P2Y_13_ receptor subtype [5]. Therefore, P2Y_13_ receptors were also contributing to calcium responses triggered by 2MeSADP in rat cerebellar astrocytes, although whether they are found as single P2Y_13_ receptors or assembling P2Y_1_/P2Y_13_ heterodimers remains unclear.

**Fig. 2:**
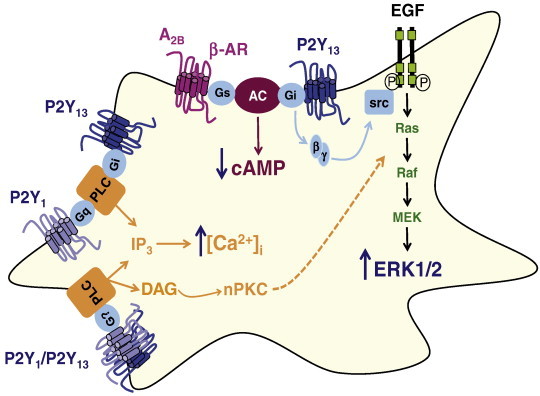
Schematic representation of the intracellular cascades triggered by 2MeSADP stimulation in rat cerebellar astrocytes. 2MeSADP can activate both P2Y_1_ and P2Y_13_ receptors, which are present in the majority of astrocyte population, and induce intracellular calcium mobilization. 2MeSADP acting through a canonical P2Y_13_ receptor, *via* Gi protein, inhibits cAMP production induced by β-adrenergic or A_2B_ adenosine receptor stimulation. Besides, βγ subunits derived from Gi proteins could be able to cross-talk to MAPK cascade activated by EGF receptors and *via* src-like kinases induce ERK activation. Besides, both P2Y_1_ and P2Y_13_ or P2Y_1_/P2Y_13_ heterodimers also activate PLC and DAG production, which could mediate nPKC activation and contribute to ERK activation.

To go deeply into the characterization of intracellular signaling coupled to P2Y_13_ receptor stimulation in rat cerebellar astrocytes, we checked one of the most important cross-talk signaling activated by Gi-coupled receptors, the activation of the extracellular regulated kinases (ERKs), which are members of the family of mitogen-activated protein kinases (MAPKs) targeted by growth factor receptors. We proved that stimulation of cerebellar astrocytes with 2MeSADP increased phosphorylation of ERK1/2, the active form of ERKs. 2MeSADP-induced ERK activation was transient, peaking at 5 min of incubation with the nucleotide and turning to basal levels one hour after treatment. ERK activation was completely prevented by *Pertussis Toxin* pre-treatment, which clearly indicated that ERK activation induced by 2MeSADP was mediated by a Gi-coupled receptor in cerebellar astrocytes, most likely the P2Y_13_ receptor. In fact, the EC_50_ value observed in ERK activation studies correlated with that obtained in experiments of cAMP production inhibition (around 40 nM) (Fig. 1C and D) [5]. Considering that P2Y_1_ receptors are present in a large population of cerebellar astrocytes, we analyzed their possible contribution to ERK activation induced by 2MeSADP. In contrast to that observed in calcium responses, ERK activation induced by 2MeSADP was insensitive to the P2Y_1_ receptor antagonist MRS2179, indicating that P2Y_13_ is exclusively mediating this response. 2MeSADP-induced ERK activation was insensitive to intracellular calcium chelation, and dependent on nPKC and src-like kinase activation. When the specific P2Y_13_ receptor antagonist MRS2211 was released to the market, we confirmed that ERK activation induced by 2MeSADP in cerebellar astrocytes was mediated by this ADP receptor subtype [unpublished results]. Fig. 2 summarizes the intracellular signaling triggered by 2MeSADP stimulation in rat cerebellar astrocytes. Current studies are revealing that P2Y_13_-induced ERK activation also displayed protective actions against genotoxic stress in these glial cells, as described below for granule neurons, and agrees well with data reported in cortical astrocytes [51].

## 3 P2Y_13_ Receptor Expression in Rat Cerebellar Granule Neurons

**Fig. 3:**
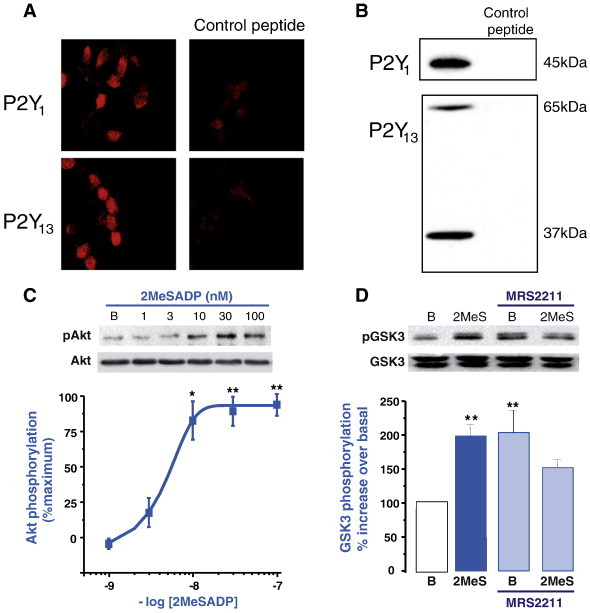
Presence of P2Y_1_ and P2Y_13_ receptors in granule neurons. A. Fluorescence images on the left show immunostaining of granule cells labeled with rabbit anti-P2Y_1_ and anti-P2Y_13_ tagged with goat anti-rabbit IgG Cy-3 conjugate. Images on the right represent different fields obtained after the incubation of P2Y antibodies with their respective control peptides. B. Expression of P2Y receptors by western blot revealed protein bands of 45 and 65/37 kDa for P2Y_1_ and P2Y_13_ receptors, respectively. P2Y antibody specificity is confirmed by the absence of signal after treatment of antibodies with control peptides. C. P2Y_13_ receptors couple to Akt phosphorylation in granule neurons. Akt phosphorylation was induced by 10 min stimulations with growing concentrations of 2MeSADP. EC_50_ values agree with the range of P2Y_13_ receptor activation and are similar to that obtained for GSK3 phosphorylation. D. GSK3 phoshorylation induced by 2MeSADP (1 μM, 10 min) in granule neurons pre-incubated for 10 min in the presence or absence of 10 μM MRS2211, the specific P2Y_13_ antagonist.

Cerebellar granule neurons constitute the major cell population of cerebellar cortex and have been widely employed in studies of intracellular signaling cascades and mechanisms responsible for cell death and survival. The presence of nucleotide receptors in cultured granule neurons has already been reported [2], describing the co-expression of several subtypes of both P2X and P2Y receptor families, and their variations according to different stages of granule cell maturation in culture. Based on intracellular calcium responses displayed by different adenine and pyrimidine nucleotides, we have identified several cell sub-populations. Cells responding to 2MeSADP were observed between 7- and 14-DIV (days *in vitro*). Among them, 40% exhibited calcium responses insensitive to the extracellular calcium chelation and the P2Y_1_ receptor antagonist MRS2179. Therefore, this kind of response could be attributed to another type of ADP-responding P2Y receptor, P2Y_12_ or P2Y_13_ subtypes [19]. Although P2Y_1_, P2Y_12_ and P2Y_13_ transcripts were expressed in granule neurons, only the P2Y_13_ receptor triggered intracellular calcium signals, as described by promiscuous coupling of this receptor to G_16_, G_i_ and Gs proteins [7,40]. Later on, the availability of specific antibodies against P2Y_12_ and P2Y_13_ receptors confirmed the presence of P2Y_1_ and P2Y_13_ proteins in granule cells. Fig. 3A depicts the specific immunostaining obtained for anti-P2Y_1_ and P2Y_13_ receptor antibodies in 10 DIV granule neurons. Moreover, western blot studies revealed specific bands for P2Y_1_ and P2Y_13_ proteins, corroborating the presence of both receptors in granule neurons (Fig. 3B).

In order to know whether P2Y_1_ and P2Y_13_ receptors were able to work in combination or elicit specific responses in this neuronal model, calcium-dependent signaling coupled to the activation of both receptors was analyzed. In this regard, we found out that only the P2Y_1_ receptor mediated the phosphorylation and activation of one of the main calcium signaling transducer, calcium calmodulin kinase II (CaMKII). This action could be observed and quantified in both soma and neurite compartments of granule neurons and was completely abolished by the presence of the specific P2Y_1_ receptor antagonist MRS2179 [35]. Moreover, we have recently reported specific functions for P2Y_13_ receptors at the level of classical pathways coupled to trophic factors, such as ERK-MAPK and glycogen synthase kinase-3 (GSK3) signaling. These evidences indicate that P2Y_1_ and P2Y_13_ receptors could be acting independently and mediating different functions in the cerebellar granule cells.

## 4 P2Y_13_ Receptors are Coupled to GSK3 Signaling in Granule Neurons

The main survival pathway present in granule neurons is the PI3K/Akt axis, which is triggered by potent trophic signals, such as the growth factor IGF-I and the neurotrophin BDNF [10,20,42,53]. One of the main targets of Akt is GSK3, which is phosphorylated in Ser^21/9^ residues (for α and β GSK3 isoforms, respectively) leading to the inhibition of its catalytic activity [17]. An increase of GSK3 activity by expression of a constitutively active form of GSK3 leads to neuronal death [20,37], whereas trophic factors maintain high levels of phosphorylated GSK3 in order to retain GSK3 in its inactive form [9,47]. GSK3 kinase activity is able to amplify several stimuli that trigger the intrinsic mitochondrial-dependent apoptotic pathway. For instance, GSK3 can phosphorylate and transcriptionally activate key pro-apoptotic factors, such as Bax and Bim, as well as to interfere with the anti-apoptotic action of Bcl-2 family proteins and CREB transcription factor [37,41].

In cerebellar granule neurons, 2MeSADP promoted a transient increase in GSK3 phosphorylation in a PI3K-dependent way. In fact, 2MeSADP was also able to stimulate the phosphorylation and activation of the upstream kinase Akt, suggesting that an ADP-responding P2Y receptor mediated the activation of the PI3K/Akt/GSK3 axis (Fig. 3C) [46]. The effect of 2MeSADP was sensitive to *Pertussis Toxin* treatment and was not modified by intracellular calcium chelation, thereby indicating the implication of a Gi-coupled receptor [46]. Additional pharmacological tools were employed to confirm this assumption, including no effect of P2Y_1_ and P2Y_12_ specific antagonists, sensitivity to P2Y_13_ antagonist MRS2211 (Fig. 3D), and similar affinities for ADP and 2MeSADP. Altogether, these data pointed out to P2Y_13_ as the receptor responsible for 2MeSADP-mediated effect on GSK3 signaling in granule neurons [46].

**Fig. 4:**
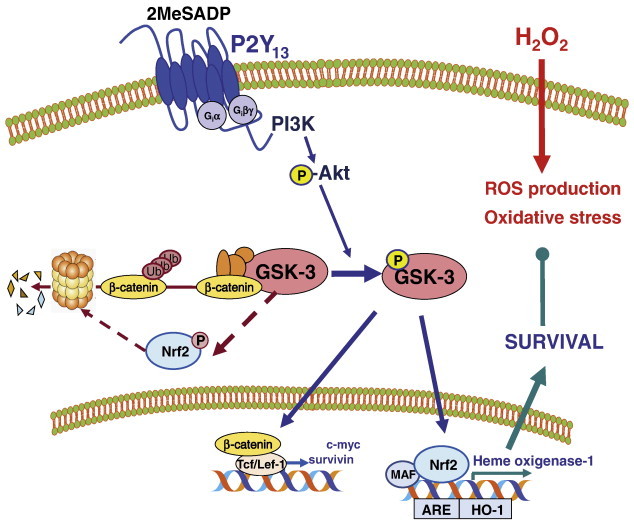
P2Y_13_ receptors couple to the survival promoting pathway of GSK3 signaling inhibition in granule neurons. P2Y_13_ receptor stimulation by the agonist 2MeSADP triggers the activation of the PI3K/Akt/GSK3 pathway in granule neurons. GSK3 phosphorylation inhibits its catalytic activity towards its substrates, allowing cytosolic stabilization and nuclear translocation of the transcriptional targets, β-catenin and Nrf2. β-catenin regulates transcription of Tcf/Lef-1-dependent genes involved in survival, differentiation and cell cycle dynamics. The transcription factor Nrf2 activates ARE-dependent genes involved in antioxidant cell response, such as heme oxigenase-1 (HO-1). Activation of the Nrf2/HO-1 axis in response to 2MeSADP stimulation in granule neurons functions as an important antioxidant defense mechanism that protects against ROS production and oxidative stress-induced apoptosis evoked by treatment with H_2_O_2_.

To shed some light on the physiological role played by P2Y_13_ nucleotide receptor, we analyzed some well-known substrates of GSK3. This enzyme is involved in the regulation of several transcription factors and modulates their function, half-life and subcellular location [17,30]. One of the best characterized is β-catenin, a transcriptional regulator that is normally associated to GSK3 in axin-containing multiprotein complexes at the cytosol. GSK3 restricts β-catenin activation by promoting its phosphorylation, which directs β-catenin to proteasomal degradation. The activation of Wnt signaling through frizzled receptors destabilizes GSK3 protein complex and releases unphosphorylated β-catenin, which enables it to translocate to the nuclear compartment and regulate transcription of Tcf/Lef-1-dependent genes. This way of GSK3 inactivation is known as the canonical pathway and is different from the PI3K/Akt-dependent pathway triggered by insulin and related growth factors. Interestingly, we demonstrated the stabilization and nuclear translocation of β-catenin following 2MeSADP treatment in granule neurons. In addition, IGF-I, which potently activates the PI3K/Akt/GSK3 axis in granule neurons, was also involved in β-catenin nuclear accumulation. Our results evidenced the presence of a cross-talk between GSK3 canonical pathway and the insulin pathway through the activation of β-catenin, and P2Y_13_ receptor as a gene transcription regulator in neuronal models [45]. Transcriptional activity of β-catenin in granule neurons has not yet been analyzed in detail, but their functions in cell cycle regulation, cell adhesion, migration, and survival have been described [18] (Fig. 4).

### 4.1 P2Y_13_ Receptor Mediated Activation of the Nrf-2/HO-1 Axis in Granule Neurons

Another interesting outcome of GSK3 signaling mediated by P2Y_13_ receptor in granule neurons involved the transcription factor Nrf2 (NF-R2-related factor-2). This factor is a master antioxidant regulator that binds antioxidant response elements (AREs) and regulates the transcription of detoxification genes. Nrf2 activation induces expression of several antioxidant enzymes of the so-called phase II response, such as heme oxigenase-1 (HO-1), providing a major mechanism in cellular defense against oxidative stress [26,32]. Nrf2 levels are low under homeostatic redox conditions, and this is achieved by its binding to the chaperone Keap-1, which retains Nrf2 at the cytosol allowing its ubiquitination and proteasomal degradation [44]. Another way of Nrf2 regulation involves its phosphorylation and translocation to the nuclear compartment. Similar to what was described for β-catenin, GSK3 acts as a negative regulator of Nrf2, which promotes Nrf2 phosphorylation and degradation restricting its transcriptional activity over inducible genes [49,50]. Any extracellular stimuli that induce GSK3 inactivation have the ability to stabilize and increase Nrf2 function. In the experimental model of granule neurons, stimulation of M_1_ acetylcholine Gq-coupled receptor activates PKC-dependent GSK3 phosphorylation that induces activation of Nrf2 and the expression of one of its target genes, heme oxigenase-1 [14].

In collaboration with Cuadrado's group, we demonstrated for the first time the coupling of a Gi-coupled receptor to the Nrf2/HO-1 pathway, which was attributable to P2Y_13_ nucleotide receptor activation. The stimulation of granule neurons with 2MeSADP led to specific nuclear accumulation of Nrf2 and the expression of its product, heme oxigenase-1, which required long incubation periods from 3 to 6 h. In addition, both Nrf2 and HO-1 expressions were in agreement with the pharmacological profile of a P2Y_13_ receptor response and were dependent on 2MeSADP-evoked inhibition of GSK3 signaling. In line with this, induction of antioxidant response elements (AREs) from the HO-1 promoter was confirmed by luciferase assays in granule neurons and in neuroblastoma N2A cells ectopically expressing hP2Y_13_ receptors [13] (Fig. 4).

The activation of this potent antioxidant defense mechanism by 2MeSADP protected granule neurons against ROS production and apoptosis induced by treatment with hydrogen peroxide. Both actions were dependent on HO-1 expression, as they were abolished in the presence of the HO-1 inhibitor protoporfyrin (SnPP). Similarly, the coupling of P2Y_13_ nucleotide receptor to the Nrf2/HO-1 axis was further reproduced in mouse granule cell cultures. As expected, in cultures obtained from Nrf2 knock-out mice (Nrf-2^−/−^), 2MeSADP failed to elicit any HO-1 expression and protection against oxidative stress [13] (Fig. 4).

This work supported the first evidence of Nrf2/HO-1 axis regulation by a nucleotide receptor that linked it directly to neuroprotection. Other examples of protection against oxidative stress were provided by cortical astrocytes, in which other ADP-responding receptor, P2Y_1_, was responsible for the survival effect through the expression of oxidoreductase genes involved in antioxidant actions [51,52].

## 5 P2Y_13_ Receptor Mediated Signaling Through ERK1/2-MAPK in Granule Neurons

Ongoing with this line of work, we next investigated other signaling cascades of key relevance in granule neurons. ERK proteins are directly involved in cell homeostasis maintained by trophic factors, such as IGF-I, GDF-15 and BDNF. These factors are coupled to ERK1/2 activation in a transient way, through a dual phosphorylation at Thr and Tyr residues by the upstream MAP kinase kinase-1 (MEK1). This signaling route contributes to survival promoting effects of trophic factors against different kinds of apoptotic stimuli, such as trophic withdrawal, exposure to excitotoxic glutamate concentrations or genotoxic stress [21,22,53].

Looking for a coincident role with growth factors it was not surprising to find ERK1/2 activation by nucleotidic agonists in granule neurons. Among them, 2MeSADP was able to induce transient ERK1/2 phosphorylation and activation, which peaked at 15 min of stimulation period. Interestingly, the pharmacological profile resembled that previously found for GSK3, since 2MeSADP-mediated ERK1/2 activation resulted to be a Gi-coupled-dependent event and also required intact PI3K activity. In addition, 2MeSADP-dependent ERK1/2 activation was only sensitive to the P2Y_13_ receptor antagonist MRS2211, once again supporting the role of P2Y_13_ as the receptor responsible for ERK1/2 signaling elicited by 2MeSADP in this cellular model. These results gave evidence that the PI3K activity was essential for P2Y_13_ receptor function in granule neurons acting as an upstream effector of both ERK1/2 and GSK3 signaling.

**Fig. 5:**
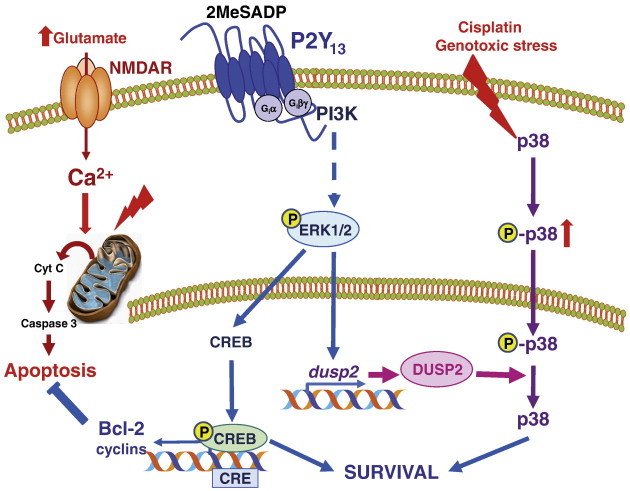
P2Y_13_ receptors activate ERK1/2 signaling and promote survival in granule neurons. P2Y_13_ receptor stimulation by the agonist 2MeSADP triggers ERK1/2 signaling in a PI3K-dependent way. Among different ERK1/2 signaling substrates, the transcription factor CREB becomes activated by phosphorylation in parallel with ERK1/2. CREB regulates expression of different genes, such as the anti-apoptotic Bcl-2, and can explain the survival promoting effect of 2MeSADP stimulation against apoptosis induced by excitotoxic concentrations of glutamate. In addition, P2Y_13_ receptor participates in cross-regulation of MAPK signaling in granule neurons. It promotes transcriptional expression of dual specificity protein phosphatase 2 (DUSP2) dependent on ERK1/2 signaling. Rise in DUSP2 protein levels is responsible of recovering basal levels of unphosphorylated form of stress related p38-MAPK, which had been activated during genotoxic stress induced by cisplatin treatment. Finally, restoring DUSP2 activity contributes to maintain cell survival in conditions of cisplatin-induced cytotoxicity.

Similarly, P2Y_13_ receptor activation partially protected granule neurons against glutamate excitotoxicity-evoked apoptosis. This survival promoting effect was dependent on the activity of CREB transcription factor, one of the main targets of ERK1/2-mediated signaling in neuronal models. Indeed, CREB phosphorylation was parallel to ERK1/2 phosphorylation following P2Y_13_ receptor activation. In addition, CREB pharmacological inhibition, not only abolished any protective effect elicited by P2Y_13_ receptors, but also severely compromised cell survival. These results agree with the role of CREB as the key regulator of the expression of genes required for long-term neuronal plasticity and suppression of apoptosis, such as the anti-apoptotic protein Bcl-2 (Fig. 5). ERK1/2/CREB-dependent survival pathway was also activated by the neurotrophin BDNF acting on TrkB receptors in granule neurons. Indeed, BDNF potently promoted ERK1/2 phosphorylation until 1 h after stimulation. In agreement with this higher level of ERK1/2 activation, BDNF behaved as a stronger effector supporting cell survival in conditions of excitotoxic glutamate concentrations [45].

### 5.1 Dual Specificity Protein Phosphatase 2, DUSP2, is an Intracellular Target of P2Y_13_ Receptor in Granule Neurons

Recently we have identified a new target of P2Y_13_ receptor mediated-ERK1/2 signaling in granule neurons, the protein phosphatase DUSP2. This is a member of the family of dual specificity phosphatases, which presents the ability to dephosphorylate both Thr/Ser and Tyr residues of MAP kinases [12,48].

Our first knowledge of dual protein phosphatases came from previous gene expression studies performed by a microarray analysis in granule neurons stimulated with 2MeSADP. Functional analysis revealed several clusters of over-represented genes related to protein phosphatase activity. It was noticeable that the concurrence of several phosphatases belonged to the family of DUSP phosphatases, particularly the protein DUSP2. QPCR experiments validated microarray results and confirmed that stimulation of granule neurons with 2MeSADP induces the transcription of *dusp2* gene within a time course that was characteristic of an immediate early gene (IEG). As expected, *dusp2* expression was abolished by the inhibition of both ERK signaling and PI3K activity, and by the P2Y_13_ specific antagonist MRS2211. These data confirmed that *dusp2* gene was under the regulation of PI3K/ERK1/2 mediated signaling stimulated by P2Y_13_ receptors in granule neurons [43].

DUSP2 belongs to a subfamily of dual specificity protein phosphatases that are specific for MAP kinases, and they are termed typical DUSPs or MKPs (MAPK phosphatases). The MKPs constitute a structural distinct group of enzymes that can be classified in different subgroups based on their substrate specificity and subcellular distribution. The group of nuclear inducible phosphatases exhibits broad specificity towards different types of MAPKs, although with some preference for the stress related kinases, p38 and JNK, being DUSP1 and DUPSP2 the most representative ones. Other types of DUSPs are constitutive and exclusively found at the cytoplasmic compartment, as is the case of the ERK1/2 selective phosphatase, DUSP6. Concerning their physiological significance, MKPs are emerging as key regulators of both the intensity and duration of MAPK signaling. Along this line, defects in DUSP expression or activity are always associated to over-activation of MAP kinases for long-periods of time, and this usually occurs during the exposition to several kinds of stress-inducing stimuli [12,31,48].

In agreement with that, we induced genotoxic stress in granule neurons by exposure to cisplatin, which is a cytotoxic drug commonly used in chemotherapy with important neurotoxic side effects. Cisplatin exposure promoted increase over time of the phosphorylated form of the stress MAPK, p38, which runs in parallel with the progressive decrease of DUSP2 protein levels. In these conditions, a previous activation of P2Y_13_ receptors had the effect of increasing DUSP2 protein expression and restoring basal phosphorylation levels of p38 (Fig. 5). As expected, P2Y_13_ receptor-mediated dephosphorylating effect on p38 as well as its prosurvival actions during cisplatin treatment were prevented by both P2Y_13_ specific antagonist MRS2211 and the inhibitor of tyrosine phosphatases orthovanadate. Therefore, P2Y_13_ activity on DUSP2 expression also contributed to neuroprotection against genotoxic stress [43] (Fig. 5).

## 6 Summary and Outlook

This work covers the present knowledge and understanding of P2Y_13_ receptor function in cell populations of the cerebellar cortex. In previous studies, P2Y_1_ expression and specific functions have already been described in both cerebellar astrocytes and granule neurons [27,5,35]. However, some ADP-activated signaling properties still remained unclear, as they were attributable to Gi-coupled receptor and not sensitive to P2Y_1_ receptor inhibition. The availability of new pharmacological tools, such as P2Y_13_ specific antagonist MRS2211, as well as specific antibodies, allowed us to ascribe these functions to the presence of P2Y_13_ receptors in both astrocytes and granule neurons.

According to the results presented here, it can be presumed that P2Y_1_ and P2Y_13_ receptors can trigger different intracellular routes, mediating diverse and independent functions in astrocytes and granule neurons. It is noteworthy that in both cell populations P2Y_1_ and P2Y_13_ receptors induce intracellular calcium mobilization. However, ERK1/2 signaling is specifically a Gi-coupled event not covered by P2Y_1_ receptor, indicating that trophic functions are mainly linked to P2Y_13_ receptors. Importantly, P2Y_13_ receptors couple ADP to neuroprotection in the neuronal model of granule cells. In this sense, they lead to the activation of transcription factors directly involved in the regulation of survival promoting genes, such as the ERK1/2-dependent target CREB. In addition, P2Y_13_ receptors also trigger the main survival PI3K/Akt/GSK3 pathway in granule neurons that is typically activated by trophic factors, activating the antioxidant defense response Nrf2/HO-1 axis that protects against oxidative stress. Therefore, P2Y_13_ receptors promote neuroprotection and increase resistance of granule cells to different kinds of apoptotic stimuli by activating both signaling mechanisms. Although extracellular ADP is not as potent as growth factors and neurotrophins in the activation of granule cell signaling and survival, it can play a pivotal role in conditions of limiting trophic factor availability.

Although the signaling mechanisms described here for P2Y_13_ receptors have been obtained from primary cultures and special caution is needed before their extrapolation to *in vivo* situation, granule neuron cultures has been accepted as an excellent *in vitro* model to study processes related to neuronal survival and differentiation. Indeed, dissociated granule cells exhibit the same dependence on trophic supply and synaptic activity to that observed during *in vivo* development and migration along the cerebellar cortex. Therefore, as it happens with other factors, such as IGF-I and BDNF, nucleotide receptors could exert similar functions *in vivo*. Evidences exist of purinergic tone at cerebellar cortex that can account for physiological responses implying nucleotide receptors. In addition, the release of high amounts of ATP after damaging or toxic conditions can produce extracellular ADP nucleotide that fully activates P2Y_1_ and P2Y_13_ receptors [8].

In other cell populations of central nervous system, P2Y_1_ as well as P2Y_13_ receptors play a pivotal role in neuronal differentiation and axonal elongation [11,59]. In addition, in the spinal cord primary neuronal cultures, both P2Y_1_ and P2Y_13_ receptors coordinate opposite regulation of glycine transport activity providing inhibition of neuronal GLYT2 and stimulation of glial GLYT1. This regulation involves a paracrine mechanism dependent on nitric oxide production and protein kinase G (PKG) activation, and supports a role of these receptors in nociception [29].

Concerning the physiological role of P2Y_13_ receptor in the non-neural tissues, it remains largely unexplored. In the red blood cells, ADP-responding P2Y_13_ receptor provides a negative feedback mechanism of ATP release to regulate plasma ATP levels [56]. P2Y_13_ receptor is also involved in the mast cell degranulation and release of antigen-induced release of hexosaminidase, whereas co-expressed P2Y_1_ receptor is responsible for intracellular calcium mobilization in response to ADP [16]. Moreover, P2Y_13_ receptor is involved in the regulation of hepatic HDL endocytosis through downstream signaling involving small GTPase RhoA and its effector ROCK1 [38]. Studies in P2Y_13_ knock-out mice revealed that they are resistant to high cholesterol diet and accentuated impaired hepatobiliary reverse cholesterol transport [36]. Therefore this work is the basis to consider pharmacological approaches to regulate HDL metabolism in dyslipidemias, one of the major risk factors of atherosclerosis and cardiovascular diseases. The P2Y_13_ activator AR-C69931MX is now under clinical development to increase cholesterol catabolism by the liver [25].

Conversely, P2Y_13_ receptor inhibition in pancreatic β-cell line is able to activate insulin release through PI3K-dependent signaling and promotes survival on pancreatic cells [3,54]. In this line, the pro-apoptotic role of P2Y_13_ receptors is also observed in the enteric nervous system, where genetic depletion of P2Y_13_ receptor resulted protective against high-fat diet neuronal loss [55].

The present work summarizes the pivotal role of P2Y_13_ receptors in the maintenance of neuronal survival against different harmful stimuli that compromise cell viability. Of relevance is the novel mechanism of action described for P2Y_13_ receptors in granule neurons that link them to protein phosphatase regulation. This study describes for the first time the participation of P2Y_13_ receptors in negative feedback regulation of MAP kinase signaling in a neuronal model. Once again, nucleotides behave similarly than trophic factors, which limit their own mitogenic signaling through the expression of different types of DUSP proteins and contribute to granule cell survival. Further studies will be necessary to determine the physiological relevance of the regulation of different types of dual protein phosphatases and whether they constitute a general signaling mechanism for other types of nucleotide receptors. P2Y-mediated regulation of protein phosphatases had been previously reported in cortical astrocytes under oxidative stress. These apoptotic conditions also evoked sustained ERK1/2 phosphorylation. The expression of protein tyrosine phosphatases (PTPs) induced by P2Y_1_ receptors was proposed as the mechanism responsible for restoring the basal levels of ERK1/2 phosphorylated form and promoting cell survival [51]. Thus, protein phosphatases could be considered as novel targets for nucleotide receptor signaling that link them to MAP kinase homeostasis and survival in different cellular models. Considering that MAPK activation can be critical in conditions related to aging and neurodegenerative diseases, DUSP proteins emerge as promising targets to restore signaling mechanisms that became deregulated in these physio-pathological conditions [23]. Further efforts are required to improve the knowledge of protein phosphatases, their regulation and activation pathways, in order to identify new pharmacological approaches.

## Conflict of Interest

The authors declare that there are no conflicts of interest.
